# Amplifications of *AURKA* and *AURKB* in a Burkitt lymphoma immunodeficiency-associated type: a case report

**DOI:** 10.31744/einstein_journal/2023RC0378

**Published:** 2023-06-21

**Authors:** Fábio Morato de Oliveira, Vinícius Gonçalves de Souza, Aparecida de Lourdes Carvalho, Fermino Sanches Lizarte, Carla Silva Siqueira Miranda

**Affiliations:** 1 Universidade Federal de Jataí Jataí GO Brazil Universidade Federal de Jataí, Jataí, GO, Brazil.; 2 Faculdade de Medicina de Ribeirão Preto Universidade de São Paulo Ribeirão Preto SP Brazil Faculdade de Medicina de Ribeirão Preto, Universidade de São Paulo, Ribeirão Preto, SP, Brazil.

**Keywords:** Burkitt lymphoma, Epstein–Barr virus infections, Aurora kinases, Interfases, HIV, Pathology

## Abstract

In equatorial Brazil, the association of Burkitt lymphoma and Epstein–Barr virus manifests at high rates. Here, we report, for the first time, amplifications of aurora kinase genes (*AURKA/B*) in a patient with a history of periodontal abscess and the presence of a remaining nodule, diagnosed with Burkitt lymphoma and Epstein–Barr virus, and /HIV positive. The patient was a 38-year-old man who presented with a 2-week-old severe jaw pain and a 3-day-old severe bilateral headache. He had a history of human papilloma virus. Interphase FISH analysis showed *AURKA* and *AURKB* amplification. The patient’s condition worsened, progressing to death a month after the initial care. Changes in the *MYCC* and *AURKA* pathways are directly associated with genomic instability. Thus, *MYCC* rearrangements and higher expression of *AURKA/B* may be associated with therapy resistance, highlighting the importance of *AURKA/B* evaluation in Burkitt lymphoma.

## INTRODUCTION

Burkitt lymphoma (BL) is an aggressive B-cell malignancy that could be associated with the Epstein–Barr virus (EBV), with a frequency of 20–30% in the sporadic type and 25–40% in the immunodeficiency-associated type. Burkitt lymphoma represents a heterogeneous group of aggressive mature B-cell malignancies.^(1)^ Burkitt lymphoma usually presents as a rapidly growing tumor and dissemination, with the primary tumor often found in the mesentery, testis, ovary, breast, kidney, and meninges. The involvement of lymph nodes, bone marrow, and the central nervous system is more common in patients with immunodeficiency.^(2)^

Genetic changes commonly found in BL include chromosome translocation with 8q24 (MYCC) and 14q32 (IgH gene), 2p12 (Ig kappa), or 22q11 (Ig lambda).^(1)^ Aurora kinase genes (*AURKA* and *AURKB*) play an important role in regulating the G2/M phase of the cell cycle and various mitotic events.^(3)^ A correlation between the amplification of aurora kinase genes and clinical aggressiveness has been demonstrated in different types of neoplasms.^(4)^ Given the aggressive response in the patient’s evolution, amplifications of *AURKA/B* genes were investigated, which may have contributed to an unfavorable prognosis. We report an uncommon case of BL immunodeficiency-associated type, with EBV, and the amplification of aurora kinase genes, in a 38-year-old patient.

## CASE REPORT

A 38-year-old man presented to the stomatology department with a 2-week-old severe jaw pain. The patient had a history of periodontal abscess that was previously drained, resulting in the presence of a remaining nodule. In addition, the patient had a severe bilateral headache for 3 days. Seven days after the initial admission, the patient returned to the service, disoriented and in poor general condition, with frontal edema, throwing up, asthenia, and hyperoxia, without fever. The patient was referred for medical attention, and a physical examination revealed right iliac fossa pain, small-volume ascites, and a bilateral medium-volume hydrocele. The patient was hospitalized for suspected pancreatitis.

**Figure 1:**
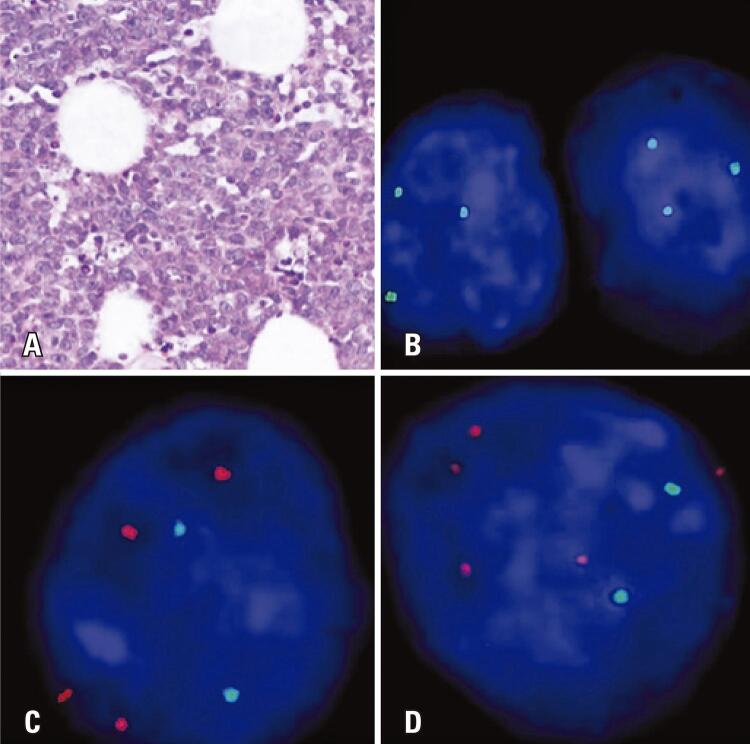
A) Burkitt lymphoma and the “starry-sky” pattern from the extranodal masses section (mesentery region); B) Interphase FISH analysis of *MYCC* gene. The *MYCC* (8q24) FISH probe is optimized to detect rearrangements of the *MYCC* gene region at 8q24 (3 spots in green) in the sample of the patient with Burkitt lymphoma; C) Interphase FISH analysis of the *AURKA* gene demonstrating elevated DNA copy number in the sample of the patient with Burkitt lymphoma (additional spots in red, control in green); D) Interphase FISH analysis of the *AURKB* gene demonstrating elevated DNA copy number in the sample of the patient with Burkitt lymphoma (additional spots in red, control in green) (Kreatech FISH Probes, Amsterdam, The Netherlands). Amplifications of *AURKA* and *AURKB* genes were observed in all analyzed cells

He had a personal history of human papillomavirus (HPV), diagnosed in 2015, and HIV, diagnosed in 2016. The latest laboratory tests showed a TCD4+ lymphocyte count of 248 cells/µL and an undetectable viral load. The patient was under treatment with efavirenz/lamivudine/tenofovir. The follow-up VDRL, HCV, and HBV tests were negative until the last visit. A biopsy in the mesentery region revealed the presence of pleomorphic lymphocytes with evident nucleoli and macrophages with clear cytoplasm and a “starry sky” appearance (Figure 1A). Immunohistochemistry was performed in an authorized diagnostic support service, revealing positivity for CD20, CD16, and BCL-6 proteins.

Interphase FISH analysis (iFISH) was performed using the following probes: *MYCC* (8q24), *AURKA*: ON *AURKA* (20q13)/20q11, and *AURKB*: *AURKB* (17/p13)/SE17 (Kreatech Fish Probes, Amsterdam, The Netherlands). *MYCC* rearrangement was easily identified via iFISH using a break-apart probe or split assay using a green probe that flanked the *MYCC* locus at 8q24 (Figure 1B), and BL was diagnosed. In addition to *MYCC* rearrangement, we performed iFISH for aurora kinase genes, and amplification of *AURKA* and *AURKB* was observed (Figure 1C and D). Cranial tomography revealed brain metastasis.

The patient had a progressive worsening of the clinical condition, progressing to death 1 month after the initial care. The patient signed a copy of the informed consent form, also signed by the main investigator, containing general information about the study and in accordance with the guidelines of the Ethics Committee (CAAE: 43920021.4.0000.5083; #4.675.519).

## DISCUSSION

Patients with BL usually display high-risk features compared to other B-cell malignancies. Endemic cases of BL are pathogenetically related to immune system disruption associated with infection by malaria and EBV. Conversely, the epidemic BL cases are associated with HIV infection, comprising 10–20% of the cases.^(5)^ In this scenario, the interactions between EBV and HIV have been closely related to BL pathogenesis. From an epidemiological point of view, patients presenting the sporadic BL form, in association with EBV, are related to poverty rates. In Brazil, the association between BL and EBV infection is more common. Usually, patients present with a symptomatic stage associated with microbiological activation of B cells.^(6)^

According to the literature, the presence of EBV confers a higher mutation rate in the lymphomagenesis process. Some data have demonstrated that EBV-negative BL arises from an early centroblast, while EBV-positive BL arises later in the development process from a memory B cell or late germinal center cell.^(6)^ In addition, the gene expression signatures of these three variants appear to be distinct, and the expression of *MYC* is an almost-universal characteristic of BL.^(7)^ In contrast, EBV-negative tumors usually present a mutation in the p53 pathway (75% of cases) that overcomes this tendency to undergo apoptosis, but these occur in only 30% of EBV-positive BL.^(8,9)^

For the first time, we identified amplifications of aurora kinase genes (*AURKA* and *AURKB*) in the sample of a patient with BL who was EBV/HIV positive. Given the unavailability of fresh material for analyzing gene expression, we performed gene amplification using the iFISH technique. Amplifications were identified in all BL cells analyzed (n=50). Amplifications of *AURKA/B* genes are normally found in many epithelial cancers and hematological malignancies. Overexpression of these genes was shown to correlate with highly proliferative and malignant cancers, poor outcomes, and low survival rates.^(10)^ The deregulation of *AURKA/B* activity or their expression in our patient may have resulted from the higher mutation rate during the lymphomagenesis process. However, it is necessary to mention that, given the patient’s early death, assessing the response to treatment was impossible.

In addition, deregulation of *AURKA/B* activity may further promote genomic instability and drive the selection of other oncogenes (*e.g., MYCC* and *TP53*), which enhance tumor invasion and metastasis, as seen in our patient (brain metastasis). We must keep in mind that patients with BL frequently present with involvement of the abdominal organs, bone marrow, and nervous system. Tumor extension could also be associated with renal function impairment and lead to metabolic disorders.^(11)^

In general, epidemic BL samples are characterized by low karyotypic complexity.^(12)^ Although it was impossible to perform a classical cytogenetic study in our patient, there is a suspicion that he could have presented with a complex karyotype, given the *AURKA/B* amplifications. To support this, some years ago, we demonstrated that *AURKA/B* overexpression was associated with genomic instability in a cytogenetically stratified group (normal *versus* abnormal karyotype) of hematopoietic cells and bone marrow-derived mesenchymal stem cells from patients with myelodysplastic syndrome.^(13)^ We also demonstrated a significant association between high expression of *AURKA* and cytogenetic profile in acute myeloid leukemia.^(14)^ *AURKA* expression is independently associated with high WBC counts. In addition, most patients with acute myeloid leukemia with overexpressed *AURKA* and *AURKB* presented with complex karyotypes.

## CONCLUSION

This case report is the first attempt to establish a relationship between *AURKA* and *AURKB* expression in a patient with Burkitt lymphoma immunodeficiency-associated type. Deregulation of the complex *MYCC/AURKA* pathway is an important event leading to genomic instability through the bypass of the G2/M checkpoints. In Burkitt lymphoma cases, expressing abnormal *MYCC* levels and high expression of *AURKA* and *AURKB* might offer some resistance to conventional therapy. Thus, aurora kinase inhibitors may also be considered for this specific subgroup of Burkitt lymphoma, whose aggressive clinical course resembles high-grade lymphomas.

## References

[1] Liu D, Shimonov J, Primanneni S, Lai Y, Ahmed T, Seiter K (2007). t(8;14;18): a 3-way chromosome translocation in two patients with Burkitt’s lymphoma/leukemia. Mol Cancer.

[2] Blum KA, Lozanski G, Byrd JC (2004). Adult Burkitt leukemia and lymphoma. Blood.

[3] Hecht JL, Aster JC (2000). Molecular biology of Burkitt’s lymphoma. J Clin Oncol.

[4] Yan M, Wang C, He B, Yang M, Tong M, Long Z (2016). Aurora-A Kinase: A potent oncogene and target for cancer therapy. Med Res Rev.

[5] Khan A, Brahim A, Ruiz M, Nagovski N (2018). Relapsed/refractory Burkitt lymphoma and HIV infection. Int J STD AIDS.

[6] Bellan C, Lazzi S, Hummel M, Palummo N, de Santi M, Amato T (2005). Immunoglobulin gene analysis reveals 2 distinct cells of origin for EBV-positive and EBV-negative Burkitt lymphomas. Blood.

[7] Piccaluga PP, De Falco G, Kustagi M, Gazzola A, Agostinelli C, Tripodo C (2011). Gene expression analysis uncovers similarity and differences among Burkitt lymphoma subtypes. Blood.

[8] Farrell PJ, White RE (2021). Do Epstein-Barr Virus mutations and natural genome sequence variations contribute to disease?. Biomolecules.

[9] Li Z, Baccianti F, Delecluse S, Tsai MH, Shumilov A, Cheng X, Ma S (2021). The Epstein-Barr virus noncoding RNA EBER2 transactivates the UCHL1 deubiquitinase to accelerate cell growth. Proc Natl Acad Sci U S A.

[10] Vader G, Lens SM (2008). The Aurora kinase family in cell division and cancer. Biochim Biophys Acta.

[11] Torchia EC, Caulin C, Acin S, Terzian T, Kubick BJ, Box NF (2012). Myc, Aurora Kinase A, and mutant p53(R172H) co-operate in a mouse model of metastatic skin carcinoma. Oncogene.

[12] Hummel M, Bentink S, Berger H, Klapper W, Wessendorf S, Barth TF, Bernd HW, Cogliatti SB, Dierlamm J, Feller AC, Hansmann ML, Haralambieva E, Harder L, Hasenclever D, Kühn M, Lenze D, Lichter P, Martin-Subero JI, Möller P, Müller-Hermelink HK, Ott G, Parwaresch RM, Pott C, Rosenwald A, Rosolowski M, Schwaenen C, Stürzenhofecker B, Szczepanowski M, Trautmann H, Wacker HH, Spang R, Loeffler M, Trümper L, Stein H, Siebert R, Molecular Mechanisms in Malignant Lymphomas Network Project of the Deutsche Krebshilfe (2006). A biologic definition of Burkitt’s lymphoma from transcriptional and genomic profiling. N Engl J Med.

[13] Lucena-Araujo AR, Oliveira FM, Leite-Cueva SD, Santos GA, Falcao RP, Rego EM (2011). High expression of AURKA and AURKB is associated with unfavorable cytogenetic abnormalities and high white blood cell count in patients with acute myeloid leukemia. Leuk Res.

[14] Oliveira FM, Lucena-Araujo AR, Favarin MC, Palma PV, Rego EM, Falcão RP (2013). Differential expression of AURKA and AURKB genes in bone marrow stromal mesenchymal cells of myelodysplastic syndrome: correlation with G-banding analysis and FISH. Exp Hematol.

